# Spontaneous healing of a ruptured pulmonary hydatid cyst: a rare phenomenon

**DOI:** 10.1590/0037-8682-0194-2022

**Published:** 2022-07-25

**Authors:** Yener Aydin, Omer Araz, Atilla Eroglu

**Affiliations:** 1 Ataturk University, Medical Faculty, Department of Thoracic Surgery, Erzurum, Turkey.; 2 Ataturk University, Medical Faculty, Department of Pulmonary Diseases, Erzurum, Turkey.

A 28-year-old female patient presented to our hospital with cough, fever, and a salty, watery expectoration. She was diagnosed with a pulmonary hydatid cyst based on thorax computed tomography and flexible bronchoscopy findings. Surgical treatment of the hydatid cyst was recommended for the patient. However, the patient did not consent to undergo surgery and medical treatment until 3 months later. Follow-up examinations revealed that the hydatid cyst cavity had completely healed (Figure 1). 

**FİGURE 1:**
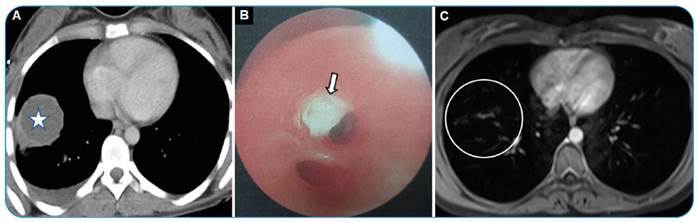
Axial computed tomography scans showing a hydatid cyst of 45-mm diameter with a unilocular water-dense lesion **(indicated with star)** and pleural effusion **(A)**. Flexible bronchoscopy findings showing a membrane **(arrow)** originating from the hydatid cyst in the anterior basal segment of the right lower lobe **(B)**. Control axial T2-weighted magnetic resonance image **(circle)** showing spontaneous healing of the hydatid cyst 3 months later **(C)**.

The lung is the second most common organ in which hydatid cysts are seen. Although a hydatid cyst is a benign lesion, it can lead to asphyxia and anaphylaxis when the cyst is ruptured. Moreover, the cyst cavity is often infected during the bronchial or pleural rupture of hydatid cysts^1^ ^,^ ^2^. Therefore, surgical treatment is indicated after the diagnosis^3^. As seen in the present case, spontaneous healing of a ruptured hydatid cyst rarely occurs. 
